# Mini Review: Effect of GLP-1 Receptor Agonists and SGLT-2 Inhibitors on the Growth Hormone/IGF Axis

**DOI:** 10.3389/fendo.2022.846903

**Published:** 2022-02-21

**Authors:** Angelo Cignarelli, Valentina Annamaria Genchi, Giulia Le Grazie, Irene Caruso, Nicola Marrano, Giuseppina Biondi, Rossella D’Oria, Gian Pio Sorice, Annalisa Natalicchio, Sebastio Perrini, Luigi Laviola, Francesco Giorgino

**Affiliations:** Department of Emergency and Organ Transplantation Section of Internal Medicine, Endocrinology, Andrology and Metabolic Diseases, University of Bari Aldo Moro, Bari, Italy

**Keywords:** growth hormone, insulin-like growth factor, GLP-1RAs, SGLT-2is, diabetes

## Abstract

Accumulating evidence supports the early use of glucagon-like peptide-1 receptor agonists (GLP-1RAs) and sodium glucose transporter-2 inhibitors (SGLT-2is) for the treatment of type 2 diabetes. Indeed, these compounds exert numerous pleiotropic actions that favorably affect metabolism and diabetes comorbidities, showing an additional effect beyond glucose control. Although a substantial amount of knowledge has been generated regarding the mechanism of action of both drug classes, much remains to be understood. Growth hormone (GH) is an important driver for multiple endocrine responses involving changes in glucose and lipid metabolism, and affects several tissues and organs (e.g., bone, heart). It acts directly on several target tissues, including skeletal muscle and bone, but several effects are mediated indirectly by circulating (liver-derived) or locally produced IGF-1. In consideration of the multiple metabolic and cardiovascular effects seen in subjects treated with GLP-1RAs and SGLT-2is (e.g., reduction of hyperglycemia, weight loss, free/fat mass and bone remodeling, anti-atherosclerosis, natriuresis), it is reasonable to speculate that GH and IGF-1 may play a about a relevant role in this context. This narrative mini-review aims to describe the involvement of the GH/IGF-1/IGF-1R axis in either mediating or responding to the effects of each of the two drug classes.

## Introduction

The development of new compounds in recent decades has substantially improved the pharmacotherapy of type 2 diabetes (T2D) by improving achievement of metabolic targets while mitigating specific side effects (e.g., weight gain, hypoglycemia) frequently observed in patients treated with traditional hypoglycemic drugs such as insulin and sulfonylureas. Among the plethora of novel agents against diabetes, GLP-1RAs are known to exert various biological effects in extra-pancreatic tissues beyond controlling hyperglycemia (1); these include a significant weight-lowering effect (−2.2 ± 2.8 kg) (2–5), which is achieved through the regulation of satiety responses *via* central mechanisms (6), and cardioprotective and renoprotective effects (7–9).

Similarly, SGLT-2is, also referred to as gliflozins, represent another class of anti-hyperglycemic agents currently used in T2D treatment with favorable effects on body weight. These medications reduce hyperglycemia (Hb1Ac reduction of −0.8 ± 1.4%) through the inhibition of glucose reabsorption in the proximal convoluted tubule of the kidney and the resulting increase in glycosuria (10–12). Notably, recent findings have described the pleiotropic effects of SGLT-2is in terms of cardioprotection as well as renoprotection (13–19).

In light of this evidence, these therapies have opened a new paradigm not only for the management of altered glucose metabolism in T2D but also in consideration of their pleiotropic effects (1, 20). Furthermore, both randomized control trials (RCTs) and real-world observational studies have reported improvements in all-cause mortality and CV mortality with both drug classes (21, 22) compared with other glucose-lowering therapies, even though the mechanisms involved have yet to be completely elucidated (23–25). Importantly, significant effects are ascribed to both medications in terms of modification of body composition involving body fat mass (26, 27), body water content (28), skeletal mass (29), and bone mineral density (30, 31), as well as the impact on the function of different organs such as the brain (32), heart (32, 33) and pancreas (5), suggesting a possible involvement of multiple endocrine axes.

Emerging data indicate that the growth hormone (GH)/insulin-like growth factor-1 (IGF-1) axis may play a role in either mediating the metabolic and neuroendocrine effect of these drugs or responding to the profound body modifications fostered by GLP-1RAs and SGLT-2is. Herein, rising knowledge on the role of GH/IGF-1/IGF-1R signaling in mediating the effect of these two antidiabetes therapies will be discussed.

## GLP-1 Receptor Agonists

### β-Cell Function

The anti-hyperglycemic benefits attributable to GLP-1RA-based therapy are primarily due to their direct action on pancreatic β-cells. Several *in vitro* and *in vivo* results have expanded the role of these molecules from potentiating glucose-stimulated insulin secretion to promoting β-cell survival under different stressful environments by favoring proliferation, neogenesis and resistance to apoptosis (34–37). In this scenario, the role of the GH/IGF-1 system in mediating the protective effect of incretin mimetics on the pancreas has been suggested, yet additional data appear to be needed. Previous studies demonstrated that the proliferative and antiapoptotic efficacy of GLP-1RAs, in particular of exendin-4, is mediated by the increased expression of IRS-2 as well as the activation of Akt, key downstream effectors of both insulin (IR) and IGF-1 receptor (IGF-1R) signaling (38). Further investigations illustrated that GLP-1 robustly stimulates IGF-1R expression and Akt phosphorylation (39), which in turn maintain pancreatic mass. The results of a transcriptomic analysis obtained from a mouse model also revealed that the stimulation of GLP-1 receptor by high doses of exendin-4 upregulates IGF-1R typically in pancreatic islets, thus reinforcing the concept that activating the IGF-1R/IRS-2/Akt pathway mediates the prevention of β-cell loss under the control of incretins (40). Furthermore, Cornu and colleagues found an autocrine loop where GLP-1 prevents the death of β-cells in an IGF-1R-dependent manner *via* secretion of IGF-2 from the same cells (39). Therefore, the activation of the IGF-2/IGF-1R axis may contribute to β-cell survival in response to GLP-1, and importantly, the increased IGF-2 secretion may be essential for triggering this response, as also observed in embryonic development (41).

In agreement with experimental studies, GLP-1 analogues, particularly liraglutide, sustain the maintenance of β-cell function in obese individuals with early T2D, and these effects are presumably independent of weight loss (42, 43). In a clinical study comparing the effects of liraglutide vs lifestyle intervention, the liraglutide group achieved a similar weight loss associated with a significant increase of IGF-2 plasma concentrations, the modifications of which were positively correlated with the β-index (43). It is tempting to hypothesize that the increased β-cell mass induced by liraglutide may be explained by the enhanced secretion of IGF-2 observed exclusively in the liraglutide arm, thus confirming that this autocrine loop ensures the recovery of pancreatic function. Interestingly, according to the weight loss and increased IGF-2 levels, patients treated with liraglutide showed changes in adipose tissue distribution with reduction of the visceral adipose tissue, which expresses IGF-2, IGF-2 receptors and IR. Hence, a possible adipo-insular axis is reasonable, where the IGF system may play a significant role (43).

Further, experimental data suggest that the pro-survival action of GLP-1RAs may also be mediated by the Akt-dependent stimulation of the mTORC1/S6K1 pathway, the activation of which is dependent upon the IGF-1R, as observed in rodent islet cells (44). Indeed, when the IGF-1R was knocked down *in vitro*, exendin-4 lost the ability to activate this pathway, suggesting that GLP-1 analogues may restore β-cell proliferation *via* autocrine or paracrine activation of IGF-1R (44).

In concert, these results define a new scenario of action for incretin-based therapy that may involve the adipo-insular axis, linking the weight lowering competence with the sustained protection of β-cells from diabetogenic stressors.

### Hypoglycemia

It is well known that GH, together with glucagon, cortisol, and epinephrine, display counterregulatory actions protecting from hypoglycemia, and that its excess may have a diabetogenic role as seen in subjects suffering from acromegaly (45). Similarly, patients with GH deficiency are at a substantially increased risk of developing insulin resistance and obesity, principally due to a lack of GH-mediated lipolytic effect on adipose tissue (46). Therefore, an involvement of the GH/IGF-1 axis in glycemic excursions associated with use of GLP-1RAs is possible. As previously mentioned, patients with T2D on incretin-based therapies showed significant weight loss and improved glycemic control with little experience of hypoglycemia (47). In this scenario, the counterregulatory action of GH may hypothetically help in promoting a euglycemic state.

In a double-blind RCT in T2D patients, a single dose of the GLP-1 analogue albiglutide did not impair the α-cell response or GH secretion when blood glucose was clamped in the hypoglycemic range (<59.4 mg/dl) (47); these results are in agreement with another study that analyzed the effects of exenatide administration (48). Similar results were also recently obtained after short-term exposure to exenatide in a cohort of patients undergoing bariatric surgery, where the amelioration of hypoglycemia was achieved *via* a mechanism not directly involving counter-regulation (49). In contrast, previous findings from Nauck et al. revealed an increase (although of small magnitude) of GH release after liraglutide therapy, suggesting that GLP-1 analogues could protect from hypoglycemic events also by activating the GH system (50).

It is well known that GLP-1 release is physiologically negligible under fasting conditions. However, when achieving pharmacological doses, conflicting data regarding the relative risk of hypoglycemic events have been noted in fasted healthy volunteers (51). Nonetheless, Lecher et al. showed the absence of hypoglycemic episodes in the presence of higher concentrations of GLP-1 when administered continuously; in this study, the Authors showed that the counter-regulatory responses in terms of changes of GH circulating levels were unaffected by GLP-1 administration during 48 h of fasting, which lead to the exhaustion of liver glycogen stores, without apparent increased risk of hypoglycemia (52).

Taken together, these data provide evidence that GLP-1RAs do not interfere with the overall counter-regulatory response to hypoglycemia both in healthy and metabolically unhealthy subjects, even though a small effect on GH secretion may be observed.

### Bone Metabolism

The GH/IGF-1 axis plays a pivotal role in the regulation of normal skeletal growth and bone health maintenance. The onset of a GH-deficiency condition leads to unhealthy bone maturation, primarily during puberty, together with reduced bone mineral density (BMD) (53). In addition, the normal bioavailability of IGF-1 is also essential for supporting a healthy bone growth throughout life, as well as for regulating bone cell growth and differentiation, and bone mineralization (54). Therefore, an imbalance of GH and IGF-1 pulses is known to increase the risk of fracture and osteoporosis, particularly in senile patients with overt metabolic disorders, such as T2DM (55). In this setting, the ability of diabetes medications to restore normal bone homeostasis is currently debated.

Bone is suggested to be a target for GLP-1, as demonstrated by knockout models in which the lack of GLP-1 receptor resulted in the development of osteopenia and bone fragility (56). However, diverging clinical results regarding the effects of GLP-1 analogues on human bone have been reported. For instance, prolonged therapy with exenatide and liraglutide did not ameliorate body BMD or serum bone turnover markers when compared to insulin (57, 58). By contrast, data from a separate pooled analysis showed that liraglutide reduced the risk of fractures differently than exenatide, which worsened bone fragility (59). Recent evidence from pilot studies has also verified that both exenatide and GLP-1 administered subcutaneously to healthy conditions favored bone resorption and remodeling without effects on circulating markers of bone formation, including IGF-1 levels (60).

Preclinical studies have also begun to elucidate specific mechanisms for the potential GLP-1-mediated bone benefits under unhealthy conditions. For instance, exenatide was found to promote osteogenesis and inhibit adipogenesis in an ovariectomized rat model (61). The GLP-1 receptor is not expressed in primary osteoblasts; hence, GLP-1 analogues may accomplish their pro-osteogenic role through other pathways. In this regard, Zhang et al. investigated, for the first time, the *in vitro* anti-osteoporotic efficacy of exenatide and noted an improvement in the proliferation rate of senescent rat osteoblasts and bone metabolism-related genes *via* IGF-1R/PI-3K/Akt signaling (62). The same Authors also demonstrated that the genetic ablation of IGF-1R abrogated the effect of exenatide on cell senescence in association with a reduction of phosphorylation of both PI-3K and Akt (62).

In conclusion, GLP-1RA could potentially exert favorable effects in the context of bone disorders in humans, largely through pro-survival and anti-senescence responses that could ameliorate bone fragility.

### Neurological Diseases

Neurodegenerative diseases are currently faced with therapeutic challenges due to the unmet need of achieving neuroprotection and improved nerve metabolism. Epidemiological studies have demonstrated that brain disorders are tightly linked to insulin resistance and T2DM (63, 64). Several factors could be involved in the progression of neurological disturbances, such as genetic and environmental agents as well as dietary habits. Moreover, the persistent condition of insulin resistance may alter the neuronal homeostatic milieu, thus exacerbating brain atrophy (65). Indeed, the sustained dysfunction of insulin/IGF-1 signaling within the central nervous system negatively impacts cell functionality and viability, thus triggering a pathogenetic cascade, as observed in Alzheimer’s disease (AD), Parkinson’s disease (PD), Huntington’s disease (HD), amyotrophic lateral sclerosis (ALS), and multiple sclerosis (MS) (66). Physiologically, both insulin and IGF-1 exert neurotrophic effects by promoting myelin sheath synthesis, astrocyte glycogen storage, oligodendrogenesis and neuronal survival (67, 68), thus guaranteeing neuronal plasticity. Therefore, the restoration of a healthy insulin/IGF-1 signaling is a significant challenge in cognitive diseases.

It is noteworthy that several antidiabetes drugs, including GLP-1RA, have shown a neuroprotective role. The existence of a gut–brain axis is already known, where GLP-1 acts both as a neurotransmitter and trophic factor by modulating cell proliferation, neurogenesis, and apoptosis (69, 70). Accordingly, a growing body of evidence has demonstrated that GLP-1RA, such as exendin-4, liraglutide, albiglutide, and lixisenatide, may cross the blood–brain barrier similarly to native GLP-1, thus potentiating neurogenesis and cognitive performance, as observed in patients suffering from PD (71, 72), as well as in a mouse model of diabetes-induced neuropathy (73). The therapeutic potential of GLP-1RA has also been verified in patients affected by multiple system atrophy (MSA), a rare neurodegenerative illness characterized by parkinsonism, cerebellar impairment, and autonomic dysfunction. The accumulation of glial cytoplasmic inclusions of α-synuclein in oligodendrocytes, impaired IGF-1R signaling, and reduced IGF-1 brain levels are the main hallmarks of MSA. Hence, modulating IGF-1 signaling could represent a strategy to antagonize the onset and progression of this disease (74, 75). It has been shown that the inadequate activity of oligodendrocytes in MSA, particularly evident in more severely affected brain regions, is principally due to insulin/IGF-1 resistance, with increased phosphorylation of IRS1(S307), which in turn inhibits the insulin/IGF-1 signaling pathway by a negative feedback loop (71). When high doses of exendin-4 were administered *in vivo* in mice, a mitigation of MSA symptoms and signs was observed in terms of motor performance amelioration, reduced load of cytoplasmic aggregates, and restoration of IRS1 phosphorylation levels in plasma neural cells (71).

HD is another motor neuron disorder characterized by a dysregulated insulin/IGF-1 signaling. HD is a rare autosomal dominant neurodegenerative pathology resulting from the neuronal accumulation of huntingtin aggregates, which subsequently lead to progressive cell dysfunction and death in the lateral hypothalamus. Several studies have also reported that neuronal disturbances in HD are accompanied by higher CNS concentrations of both GH and IGF-1, the levels of which are linked to the severity of pathology, as observed in both humans (76) and a mice model (77); on the other hand, an enhanced activation of the insulin/IGF-1 pathway produces mitochondrial dysfunction and oxidative stress. These pathological signs were alleviated after liraglutide treatment, which reduced both insulin and IGF-1 brain levels together with ameliorating whole-body energy balance (77).

Promising results also derive from a brain ischemia-reperfusion injury model, where the effects of GLP-1 (9-36), a peptide resulting from cleavage of GLP-1 (7-36) by dipeptidyl peptidase-4, were investigated. When administered systemically, GLP-1 (9-36) facilitated the functional recovery of stroke-dependent cerebral damage, while these responses were blunted in the presence of IGF-1R deficiency (78). Moreover, the GLP-1 (9-36)-induced neuronal benefits were also evident *in vitro*, with this peptide alleviating the hypoxia-dependent inflammation of astrocytes by reducing NF-κB-p65 expression. However, the anti-inflammatory property of GLP-1 (9-36) was lost in astrocytes with genetic deletion of IGF1-R, thus highlighting the key role of IGF1-R activation in these protective effects (78). The neuroprotective efficacy of incretin-based drugs also emerged in a recent study by Zhang et al., showing that exendin-4 therapy resulted in reduced infarct volume and neuronal recovery in the mouse brain by upregulating IGF-1R together with sustained activation of the PI3K/AKT/mTOR/HIF-1α pathway (79).

Together, these results provide experimental evidence that both hyperstimulation and downregulation of IGF-1R signaling may have a pathogenetic role in the onset and/or progression of neuronal disorders. In this scenario, novel frontiers open regarding the extra-pancreatic effects of GLP1-RAs, making them suitable candidates for treating these brain conditions at least in part *via* restoration of CNS IGF-1 signaling.

### Heart

Current knowledge supports the cardioprotective role of GLP-1RAs and their ability to favor functional recovery after myocardial infarction. Early clinical (80, 81) and experimental (80, 82, 83) evidence supports the role of this drug class in the repair and remodeling of the injured myocardium. After heart ischemia, a dynamic process begins that involves a cascade of extracellular matrix deposition and the prolonged production of collagen until the formation of a myocardial scar. Under this setting, GLP-1 appears to hinder the fibrotic process and stimulate the deposition of new elastic fibers that can alleviate scar stiffness, thus leading to a functional tissue recovery. To date, the paucity of data regarding the mechanisms underlying the GLP-1-induced cardio-protection makes it difficult to understand the role of these agents in the restoration of post-injured myocardium tissue. Nevertheless, recent *in vitro* findings have elucidated that both active and apparently bio-inactive forms of GLP-1 activate elastogenesis in cardiac fibroblasts in a PI-3K/Akt-dependent manner *via* IGF-1R cross-activation (84). Therefore, these experimental results, despite being preliminary, indicate that the cardioprotective role of GLP-1 may be due, at least in part, to its elastogenic property, which depends on activation of the IGF-1R pathway.

## SGLT-2 Inhibitors

### Muscle

Huang et al. (85) examined the hormonal changes after SGLT-2i therapy in a mouse model of obesity. After dapagliflozin administration, obese mice showed a significant reduction of insulin levels, lipid content and inflammation of adipose tissue and liver, and increased muscle mass. The metabolic improvement was associated with enhanced pulsatile GH secretion with no changes in pulse frequency. In addition, a full restoration of IGF-1 gene expression occurred in muscle, albeit its circulating levels were not modified, thus suggesting that IGF-1 could exert a protective role on lean mass preservation in an autocrine/paracrine manner (85). As mentioned, obesity and diabetes are associated with chronic systemic inflammation, which also involves skeletal muscle. In this setting, canagliflozin-based therapy, beyond weight lowering efficacy and amelioration of body fat distribution and hepatic steatosis, may alleviate muscle inflammation by decreasing proinflammatory markers. In addition, SGLT-2i enhanced IGF-1 expression, which protects against muscle mass loss and promotes recovery of contractile force, as observed in a mouse model of obesity (86).

A key consequence of diabetes-associated hyperinsulinemia is the sustained activation of the IGF-1R signaling pathway. At a cellular level, this condition results in an increased production of reactive oxygen species (ROS), which may induce cell senescence *via* p53 (87). The hyperactivation of insulin/IGF-1 signaling also favors cell senescence through other pathways, such as the inhibition of P1-3K and SIRT1, which provide a downstream activation of the p53-p21 pathway (88, 89). In this scenario, SGLT-2is may promote cellular repair mechanisms and prevent cells from being exposed to oxidative stress by upregulating energy deprivation sensors (e.g., AMPK, SIRT1, etc.), as well as by inhibiting insulin/IGF-1 signaling, ultimately leading to decreased circulating glucose and amino acids. Therefore, SGLT-2is appear to mimic nutrient deprivation states, thus attenuating cellular ageing and stress, and to alter cellular metabolic programming through a dormancy state with increased production of ketone bodies (90).

### Ketosis

Nutrient deprivation results in decreased blood glucose levels with consequently reduced insulin circulating levels together with an increase of counter-regulatory hormones, such as glucagon and GH. Under this condition, the first source of glucose is glycogenolysis. When fasting periods are prolonged (> 24–60 h), FFAs represent the key fuel of whole-body energy homeostasis. Under these conditions, the increased secretion of counterregulatory hormones stimulates FFAs oxidation in the liver and accelerates the production of ketone bodies, which are metabolized in muscle, adipose tissue and, most importantly, in heart and brain (52).

As demonstrated in a recent meta-analysis, SGLT-2i treatment is associated with increased plasma fasting glucagon levels in patients with diabetes compared with non-SGLT-2i treatments with a reduction of insulin and increased ketone body levels (91). Noteworthy, diabetic ketoacidosis has been reported in patients with undiagnosed active acromegaly, as demonstrated in a recent case series (92). Hence, a role for GH in stimulating ketosis can be reasonably postulated. Indeed, de Rocha et al. observed that SGLT-2i induced ketogenesis *via* GH upregulation. In particular, the administration of empagliflozin in individuals with prediabetes or diabetes was associated with the amelioration of glycemia and insulin sensitivity and with increased β-hydroxybutyrate (BHB) levels. However, when empagliflozin was administered with pegvisomant, a GH receptor antagonist, BHB levels were restored to baseline; thus, it was suggested that SGLT-2i therapy may promote ketogenesis by inducing GH (93). Therefore, diabetic patients with acromegaly could be treated with SGLT-2i, albeit caution is required for patients with uncontrolled disease.

## Conclusions

**Figure 1:**
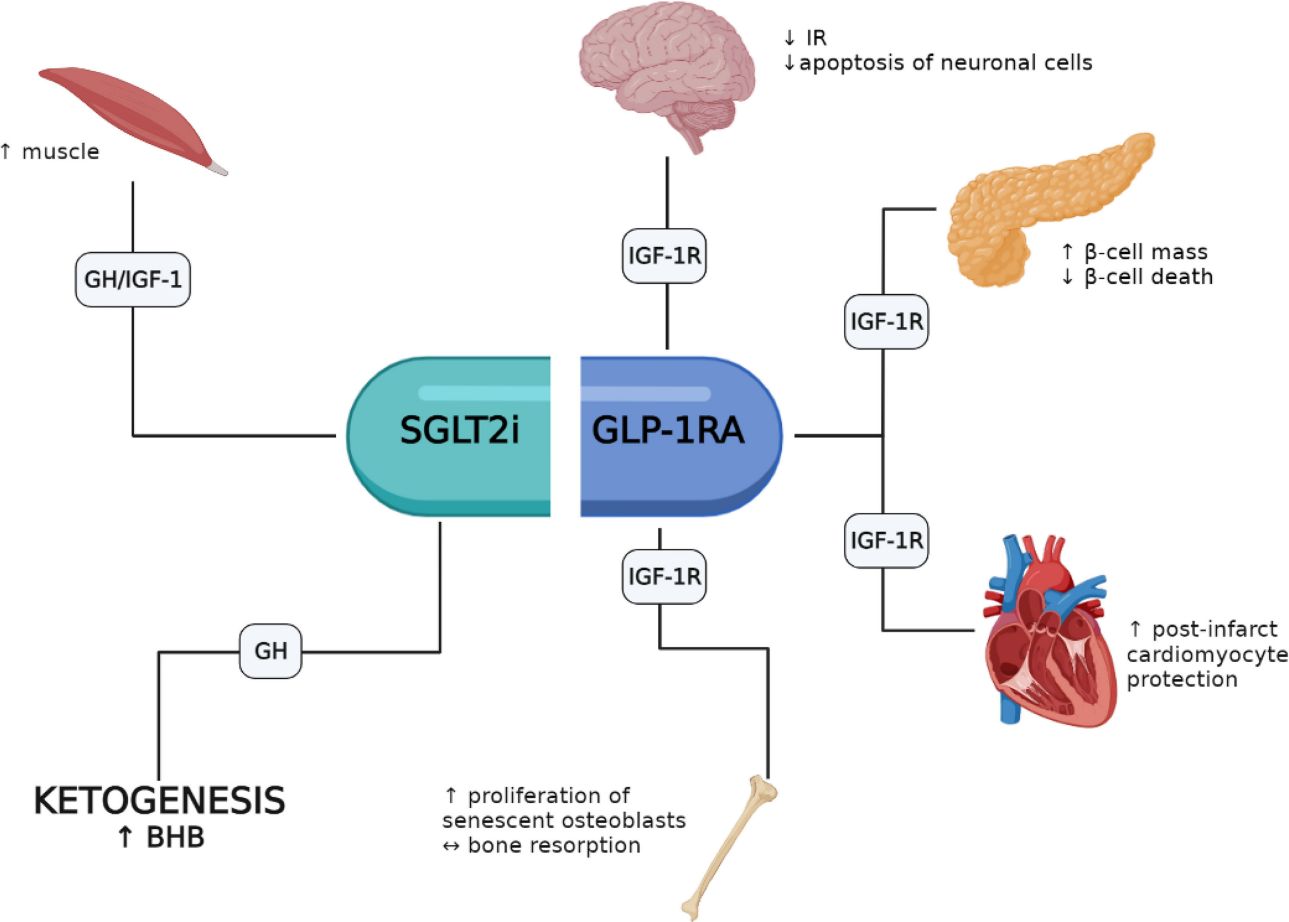
Potential involvement of the GH/IGF-1/IGF-1R axis in the pleiotropic effects elicited by GLP-1RAs and SGLT-2is.

A growing body of evidence indicates that several of the pleiotropic responses elicited by GLP-1RAs and SGLT-2is involve the activation of the GH/IGF-1/IGF-1R system. GLP-1RAs appear to favor pro-survival responses in β-cells, heart, brain and bone where the IGF-1/IGF-1R system appears to play an important role. Conversely, fewer studies have investigated the role of SGLT-2is on GH/IGF-1; yet these agents may favor an increase of lean mass and mimic an energy deprivation state with increased ketogenesis (i.e., BHB) through increased circulating GH levels (**Figure 1**). Noteworthy, the association of GLP-1RAs and SGLT-2is is representing a new pharmacological opportunity opening an innovative scenario with potential additive pleiotropic effects that will require further studies. Moreover, patients with acromegaly treated with either GH-receptor antagonists or somatostatin analogues represent a peculiar setting that need to be studied in depth to define safety and efficacy of both drug classes and their interaction with the GH/IGF-1 axis. However, it should be recalled that most results obtained so far derive from preclinical studies, and *in vivo* investigations in humans are thus necessary to fill this gap.

## Author Contributions

AC and GLG contributed to conception of the review. GLG retrieved the main articles included in the review. AC, VAG, and GLG wrote the first draft of the manuscript. FG supervised the project and finalized the manuscript. All authors contributed to manuscript revision, read, and approved the submitted version.

## Conflict of Interest

FG received research support from Eli Lilly, LifeScan, and Takeda and served as a consultant and author for AstraZeneca, Boehringer Ingelheim, Eli Lilly, Merck Sharp and Dohme, Novo Nordisk, Roche Diabetes Care, and Sanofi.

The remaining authors declare that the research was conducted in the absence of any commercial or financial relationships that could be construed as a potential conflict of interest.

## Publisher’s Note

All claims expressed in this article are solely those of the authors and do not necessarily represent those of their affiliated organizations, or those of the publisher, the editors and the reviewers. Any product that may be evaluated in this article, or claim that may be made by its manufacturer, is not guaranteed or endorsed by the publisher.
